# Exploring staff and students’ understanding and experience of Public and Patient Involvement (PPI) in an Irish University

**DOI:** 10.1186/s40900-026-00896-3

**Published:** 2026-06-23

**Authors:** Ben Kavanagh, Stacey Grealis, Damian Haslett, Patricia M. Kearney, Larry Masterson, Stephanie Skeffington, Emmy Racine, Carol Kelleher

**Affiliations:** 1 PPI Ignite Network@ UCC and School of Public Health, University College Cork, Cork, Ireland; 2 PPI Partner, PPI Ignite Network@ UCC, University College Cork, Cork, Ireland; 3 Institute of Social Science in the in the 21st Century (ISS21), University College Cork, Cork, Ireland

**Keywords:** Public and Patient Involvement, Public involvement, Participatory research, Lived experience, Irish research, PPI support, PPI training

## Abstract

**Aim:**

The aim of this study was to explore staff and students’ understanding and experiences of Public and Patient Involvement (PPI) at University College Cork (UCC), taking into account the university’s recent establishment of a dedicated research unit to advance PPI capacity. The study sought to identify the barriers, facilitators, and opportunities for strengthening and embedding high-quality PPI within the university’s research practices.

**Methods:**

A cross-sectional survey was distributed via email and social media to all UCC staff and students. The 18-item survey, informed by prior instruments and refined with PPI and stakeholder input, captured quantitative and qualitative data relating to PPI understanding, awareness, satisfaction, motivation, barriers, and enablers. Responses were analysed using descriptive statistics and content analysis.

**Findings:**

A total of 289 respondents took part in the survey, of whom 69.1% (186/269) were staff members and 30.9% (83/269) were students. Analyses were based on the number of respondents who answered each item. Overall, 64.4% (168/261) rated their PPI knowledge as reasonable, good, or very good, supported by their ability to correctly identify authentic examples of PPI. Among those familiar with PPI, just over half (51.6%, 98/190) had implemented it in their research. The top reported barriers were funding (50%, 81/162), time constraints (48.8%, 79/162), and concerns about tokenistic engagement (42.6%, 69/162). Content analysis of free-text responses highlighted the need for both structural and cultural changes to enable meaningful PPI, including institutional recognition and integration, improved resourcing and funding, fair treatment and remuneration, and cultural and attitude change. Respondents also identified three priority areas for future training: 1. PPI recruitment and facilitation, 2. Managing and communicating with contributors, and 3. Securing funding for PPI activities.

**Conclusion:**

This study demonstrates that while UCC staff and students report a generally strong understanding of PPI, this level of understanding and implementation varies by department. Practical implementation remains limited and constrained by barriers such as time, funding, and tokenism. Addressing these challenges requires institutional commitment, resources, and cultural change. Prioritising targeted training alongside structural supports offers clear opportunities to strengthen capacity and embed meaningful PPI within the university research environment.

**Supplementary Information:**

The online version contains supplementary material available at 10.1186/s40900-026-00896-3.

## Abstract (de)

Public and Patient Involvement (PPI) means doing research with or by members of the public, rather than to, about or for them. Although many people agree PPI is important in health and social care research, it is not common practice. This study looked at staff and students’ understanding and experiences of PPI at University College Cork (UCC), Ireland. A survey was shared by email and social media. The survey contained questions relating to PPI understanding, awareness, satisfaction, motivation, barriers, and enablers. The survey received responses from 269 individuals, 70% of the people who answered the survey were staff members and 30% were students. The results of the survey show that almost 70% said they had a good understanding of PPI, but only half of these people had used it in their research. The biggest barriers were lack of time, funding constraints and worries about tokenistic involvement. Survey respondents said the University could help by recognising PPI as an important part of research, providing more funding and resources, ensuring fair treatment and payment for PPI contributors, and encouraging positive attitudes to PPI. Respondents also identified three important areas for future PPI training: 1. PPI recruitment and facilitation, 2. Managing and communicating with PPI contributors, and 3. Securing funding for PPI activities. Overall, the findings show that while respondents have a good understanding of PPI, more support and cultural change are needed to make it a routine part of research at UCC.

## Background

PPI is a key principle in health and social care research, ensuring that the voices of patients, service users, carers, and the wider public help shape decisions about research priorities, design, conduct, and dissemination [1]. Defined as research that is carried out ‘with’ or ‘by’ members of the public, rather than ‘to’, ‘about’ or ‘for’ them [1], PPI has been shown to enhance research relevance, quality, efficiency and value [2, 3].

Although the importance of PPI in health and social care research is widely acknowledged, it has yet to become routine practice. For example, an analysis of 3,000 papers published in *BMJ Open* in 2022 found that only 20.6% reported PPI involvement [4]. While previous research has identified common barriers and enablers to PPI, and awareness and capacity building are often cited as key to improving implementation [5], significant capacity gaps remain [6]. Previous research highlights the importance of ensuring that training and capacity building activities are not generic but are specifically responsive to the actual needs- be they individual, organisational or systemic of the intended participants [7, 8].

Therefore, the aim of this study was to explore university staff and students’ understanding and experiences of PPI, and to identify the barriers, facilitators, and opportunities for strengthening capacity, and embedding high-quality PPI within health and social care research at UCC.

The current study employed a strong PPI Partnership (see methods), with the aim of ensuring that the survey, results, and interpretation of the findings were shaped not only by academic researchers but also by individuals with lived experience and expertise of PPI contribution, thereby improving the relevance and accessibility of our research.

## Method

### Setting

This study was undertaken in UCC, Ireland. In Ireland, a National PPI Ignite Network was established in March 2021 to promote excellence and inspire innovation in PPI within health and social care research. Funded by the Health Research Board and the Irish Research Council, with additional support from the seven lead site universities including UCC, University of Limerick, University of Galway, Trinity College Dublin, University College Dublin, Dublin City University, and RCSI University of Medicine and Health Sciences. The network is made up of academic staff and researchers from the aforementioned universities who work in partnership with over 70 national, local and charitable organisations. These partnerships allow individuals with lived experience of health conditions, carers and representatives from community and patient organisations to get involved in shaping priorities, and advising and engaging in the development of research practices in Ireland [9]. PPI Ignite Network@ UCC was established in March 2021 with the overarching aim to build capacity for high quality PPI within UCC and amongst wider society, and to embed PPI within its institutional policies and practices.

### Design

A cross-sectional online survey was distributed via email to 19,000 staff and students at UCC. The survey was constructed and distributed using the online software ‘Qualtrics’ and captured quantitative and qualitative data.

### Survey instrument

Survey questions were initially informed by unpublished PPI surveys previously conducted by the Health Research Board and other PPI Ignite Lead Sites. These surveys explored similar topics including researchers and students’ interest in PPI, understanding of PPI, motivation for conducting PPI, and barriers and enablers to PPI [5] To ensure that the survey was relevant across a wide range of disciplines, representatives from the four UCC colleges (College of Medicine and Heath, College of Arts Celtic Studies and Social Sciences, College of Science, Engineering and Food Science, College of Business and Law) were also asked to review and provide feedback on the information sheet and survey questions. Based on this feedback, some questions were re-worded to reduce emphasis on health and clinical research and to ensure broader applicability. The survey was reviewed and piloted by our PPI partners whose feedback led to further revisions of the survey instrument (See Public and Patient Involvement Section). In addition to demographics, the final survey addressed three primary research questions:

1. What are UCC staff and students’ understandings of PPI?
2. What are staff and students’ experiences of conducting PPI at UCC?
3. What are the barriers and enablers to PPI, and how can UCC develop PPI in research?

The 18-item survey was divided into four sections: (1) Demographics, (2) Understanding of PPI, (3) Experience of PPI, and (4) Barriers and enablers to conducting PPI (see Appendix A). A summary of the survey instrument questions can be found in Supplementary Table 1 (online).

### Respondents and data collection

The survey was distributed through UCC email databases and via social media (X, *formally Twitter*). Responses were predominantly obtained through the emailed link (98%). To be eligible to complete this survey, respondents must have been either a member of staff or a student at UCC. Respondents were invited to participate in the survey through several UCC mailing lists such as ‘student surveys’ (approx. 15000 recipients), ‘all academic and research staff’ (approx. 4000 recipients), and the PPI Ignite Network@ UCC’s mailing list. In total the survey was distributed to approximately 19,000 staff and students. Based on previous response rates seen with surveys of this kind in Irish universities, a goal of 350 responses was established (1% response rate from students, 5% response rate from staff). The survey remained open to responses for 6 weeks.

Survey items were not mandatory to reduce respondent burden and to accommodate the heterogeneous demographic composition of our sample. We anticipated responses from individuals with varying experience and familiarity with research and PPI itself. Allowing non-mandatory items may also improve data quality by reducing the likelihood of arbitrary responses given solely for survey continuation [10].

### Data analysis

Once the survey closed to new responses, the data was exported from Qualtrics (2022) to Microsoft Excel (Excel for Microsoft 365). The dataset was screened for duplicate responses prior to analysis, none were identified. For quantitative responses, Qualtrics and Microsoft Excel were used to calculate frequencies and descriptive statistics, and to generate graphs. Qualitative data from free text responses were copied into a Microsoft word document. Free text responses were analysed using content analysis [11]. Similar responses were grouped and systematically assigned descriptive labels using an inductive approach. Participants’ free text responses regarding their understanding of PPI were categorised into three levels: ‘excellent understanding of PPI’, ‘good understanding of PPI’, and ‘minimal or no understanding of PPI’.

The data used in this study cannot be shared publicly due to the conditions of the original ethics approval, which limited access to the research team only. Because data sharing was not included in the ethics application, retrospective approval cannot be obtained. All analyses are based on the full dataset, and all relevant results are reported in the manuscript.

### Public and patient involvement

Two PPI partners (SG and SS) were involved from the outset of this study. They participated in early discussions on study design and reviewed and piloted the initial draft of survey questions. Their input resulted in several key changes, including ensuring anonymity by not collecting personal identifiers, revising the survey information sheet, and refining the wording, order, and format of questions. They also suggested including a short introduction to PPI and a diagram of the research process at the beginning of the third survey section (after respondents were asked about their understanding) to support respondents’ understanding of PPI prior to completing the following survey items. PPI partners also suggested separate additional responses for barrier and enabler questions (Q15, Q16). The response option ‘I am not aware of any barriers/enablers’ was added to distinguish between respondents who had PPI experience but perceived ‘there are no barriers/enablers’ as opposed to those with no/limited PPI experience. After data collection closed, two meetings were held with PPI partners (SS, SG, LM) to review the raw dataset and identify priority findings for inclusion in the manuscript. All three partners subsequently reviewed draft versions of the manuscript and are co-authors on this publication.

Equality, diversity and inclusion (EDI) guidelines for PPI were followed to support meaningful and collaborative engagement of PPI Partners and Academic researchers throughout the research process [12]. PPI was reported in accordance with the Guidance for Reporting Involvement of Patients and the Public (GRIPP2-SF) checklist. The authors agree that PPI involvement enhanced the accessibility and readability of the survey instrument and the and clarity of the final manuscript.

### Ethics

Full ethical approval for this study was granted by the Social Research Ethics Committee (SREC) at University College Cork (Log 2023-067). Participation in the study was voluntary, and written informed consent was obtained from all respondents prior to survey participation. Respondents were provided with information about the purpose of the study and what participation involved prior to participation. All data were collected anonymously.

## Results

### Section 1: Demographics

The survey was open to responses for six weeks from the 6th of June to the 18th July, 2023. The survey was initially accessed by 289 respondents, although some items had a high non-response rate. Table 1 provides an overview of respondent demographics. Staff members represented the majority (69.1%) compared with students. Respondents held a variety of positions across the university, with lecturers (16.7%) and undergraduate students (16.4%) being the most common respondent. When asked to indicate their affiliation, almost half (49%) were affiliated with the College of Medicine and Health, with the remaining distributed across the other three UCC colleges. Content analysis of the free text ‘other’ responses predominantly indicated affiliation with administrative services and other research institutes and centres.

**Table 1:** Participant characteristics

Category	*N*	%
**Gender**	**269**	**100%**
Female	208	77.3%
Male	54	20%
Other / Prefer not to say	7	2.6%
**University Staff**	**186**	**69.1%**
*Academic*	*65*	*24.1%*
Lecturer	45	16.7%
Senior Lecturer	13	4.8%
Professor	7	2.6%
*Research*	*39*	*14.5%*
Research Assistant	15	5.6%
Postdoctoral Researcher	19	7%
Research Fellow	5	1.9%
*Support/Other*	*82*	*30.5%*
General Administrator	32	11.9%
Research Support Officer	29	10.8%
Research Administrator	9	3.3%
School/ Dept Manager	4	1.5%
Other / Not specified	8	3%
**University Students**	**83**	**30.9%**
Undergraduate Student	44	16.4%
Master’s Student	15	5.6.%
PhD Student	24	8.9%
**Primary Affiliation**	**267**	**100%**
College of Medicine and Health	131	49%
College of Arts, Celtic Studies, & Social Sciences	54	20.2%
College of Science, Engineering, & Food Science	29	10.9%
College of Business & Law	17	6.3%
Other	36	13.5%

### Section 2: Understanding of PPI

#### Self-rated PPI understanding (Q.4)

Of the respondents, 16.1% (42/261) self-rated their understanding of PPI as ‘very good’, 32.2% (84/261) as ‘good’, 21% (55/261) as ‘reasonable’, 16.1% (42/261) as ‘limited’ and 14.6% (38/261) reported that they had no understanding of PPI.

#### Descriptive responses to understanding (Q.5)

A free text question assessed respondents level of understanding of PPI. Responses were categorised in three groups: Excellent understanding of PPI (30.5%, 47/154), good understanding of PPI (52%, 80/154), or minimal/no understanding of PPI (17.5%, 27/154). See Table 2. Appendix B provides an overview of the free-text responses to Q.5.

**Table 2:** Descriptive responses to understanding (Q.5)

#	Category	Criterion	Example Quote
1	Excellent Understanding of PPI	Detailed descriptions of their understanding of PPI and referenced involvement in research at all stages of a research study. Noting a partnership and understanding that PPI is co-produced research going beyond tokenistic engagement was also evident, as this response exemplifies	*‘Doing research with patients*,* public*,* members of the public (those for whom the research may have an impact) rather than conducting research on people. It is bringing stakeholder voices into decision making and activities at all stages of the research from design to dissemination and translation.’(Lecturer)*
2	Good understanding of PPI	Provided correct definitions/understanding of PPI and the factors associated with it. However, they lacked detail which could potentially demonstrate a more nuanced understanding	*‘Doing research “with” or “by” partnering with patients and the public. The PPI group are active collaborators on the study/project and NOT participants.’ (Postdoctoral Researcher)*
3	Minimal or no understanding of PPI	Provided minimal input in relation to what PPI consists of or provided incorrect definitions of PPI. A common misconception evident among these respondents was that PPI consists simply of participation in research by public and patients rather than involvement in the research design	*‘Patient information policy*,* and public transparency.’ (Research Assistant)*

#### Application of understanding of PPI (Q.6)

Respondents (*n* = 200) were asked to ‘select all that apply’ from a list of actions which they considered to be PPI. The two most common selections were ‘inviting members of the public, patients or carers to sit on a study/research group steering committee’ (78.5%, 157/200), and ‘working with members of the public and other stakeholders to set research priorities’ (78%, 156/200), both of which are examples of PPI. Similarly, the options which had the least engagement from respondents were ‘recruiting study participants to take part in a study’ (32%, 64/200), and ‘conducting qualitative interviews, where researchers interview study participants’ (33%, 66/200) which are examples of non-PPI research activities.

This suggests that respondents in general were able to appropriately identify a PPI activity and confirmed the self-rated understandings. For example, half of the respondents who self-reported as having had ‘no understanding of PPI’ incorrectly identified ‘recruiting study participants to take part in a study’ as a PPI activity.

### Section 3: Experience of PPI

From a total of 190 responses to Q.7, half of the respondents (51.6%, 98/190) reported engaging in PPI in their own research, 13.2% (25/190) stated that they always included PPI in their research, while 38.4% (73/190) reported sometimes including PPI in their research, and 48.4% (92/190) reported having never included PPI in their research. Of the staff who responded to Q.7 (*n* = 137), 62.8% indicated either always or sometimes including PPI in their research. In contrast, of students who responded to Q.7 (*n* = 53), 22.6% indicated either always or sometimes including PPI in their research. A general trend of increasing PPI experience with increasing academic seniority was observed. Among students, 25% (*n* = 28) of undergraduate students, and 31.6% (*n* = 18) of PhD students reported including PPI in their research while no master’s students (*n* = 7) indicated PPI involvement. In contrast 70.6% (*n* = 34) of lecturers, 100% (*n* = 12) of senior lecturers and 100% (*n* = 6) of professors reported including PPI in their research. Notably, the number of responses varied substantially across roles. Those with no experience of PPI were instructed to skip to Q.13.

There was a high level of satisfaction amongst respondents who had previously included PPI in their research with 87.8% (86/98) reporting they were either very satisfied or somewhat satisfied with PPI, while no respondents reported being very dissatisfied (Q.8).

Table 3 below shows the stages of the research process that respondents (*n* = 90) have involved PPI contributors (Q.9). Overall, PPI was spread across multiple stages of the research process with study recruitment (61.1%, 55/90) and study design and procedures (58.9%, 53/90) being the most common selected responses. Fewer respondents reported involvement in evaluation and implementation stages.

**Table 3:** Stages of the research process that PPI was conducted (Q.9)

#	Stages	*N* (*n* = 90)	%
1	Study recruitment e.g. developing research subject recruitment procedures and materials	55	61.1
2	Study design and procedures e.g. developing research instruments, choosing primary research outcomes, finalising research protocols	53	58.9
3	Dissemination e.g. presentations, manuscripts, social media, plans for future studies	47	52.2
4	Data collection e.g. public or patient administered data collection such as interviews or surveys	46	51.1
5	Agenda setting and funding e.g. generating ideas, identifying research topics, prioritising research topics, funding applications, protocol preparation	44	48.9
7	Data analysis e.g. interpretation of findings, external review	33	36.7
6	Evaluation e.g. evaluation of process measures, adherence and uptake of interventions, plans for future research	33	36.7
8	Implementation e.g. developing decision aid tools, developing clinical practice guidelines	21	23.3
	Other	5	5.6

Respondents (*n* = 91) primary motivation for including PPI in their research was to improve the quality of research (82.4%, 75/91), followed by researchers feeling that it was a ‘good idea/right thing to do’ (71%0.4, 65/91), PPI being a requirement to achieve funding as their motivation was also commonly cited (37.4%, 34/91) (Q.10).

Of those with PPI experience, 70% of survey respondents (62/89) reported that they had received no training for PPI, and 30% of survey respondents (27/89) had received some training (Q.11). Those who received PPI training received it through groups such as PPI Ignite Network@ UCC and the Evidence to Support Prevention Implementation and Translation (ESPRIT) research group among others. Outside of UCC, respondents had received training from the Health Research Board and from the PPI Ignite network (e.g., PPI Summer School).

Of those with PPI experience, 55.8% (48/86) reported receiving advice, while 44.2% (38/86) reported that they had received no advice in this area (Q.12). PPI Ignite Network@ UCC was cited as the main source of advice and support for research involving PPI, noting that support for carrying out PPI activities was provided through workshop, training events and in lectures/lecture material on research methods.

There was very strong interest in involving PPI in prospective research with 65.5% (114/174) noting that they are interested in involving PPI in their future research, 27.6% (48/174) noted that they would be interested in PPI providing that the adequate support was available and 6.9% of respondents (12/174) stated that they could not see the benefit of integrating PPI into their research (Q.13). Respondents who had not considered carrying out PPI to date were asked to outline their reasons why. Content analysis of the free text responses (*n* = 53) identified five general reasons:1. Perceived Irrelevance to their Role and/or Research Area, 2. Lack of Awareness or Understanding, 3. Structural, Practical and Institutional Barriers, 4. Lack of Training, Support, and Tools, 5. Future Intentions to embed PPI. See Appendix C for further details (Q14).

### Section 4: Barriers and enablers

#### Barriers

Funding, time constraints, and concerns about tokenistic engagement were the most widely cited barriers to PPI by respondents (see Table 4 below). While both staff and students reported funding and time constraints as the most significant barriers, staff (*n* = 118) more often noted concerns about tokenistic engagement as an additional key barrier, whereas students (*n* = 44) highlighted difficulty recruiting patients and members of the public.

**Table 4:** Barriers to involving patients and members of the public in the design, conduct and dissemination of research (Q.15)

#	Barrier	*N* (*n* = 162)	%
1	Funding	81	50
2	Time	79	48.8
3	Concerns about tokenistic engagement	69	42.6
4	Recruiting patients and members of the public who are interested	57	35.2
5	I have found it difficult to pay PPI contributors	47	25.8
6	Concerns about not being able to meet patient or public expectations	41	25.3
7	I don’t know how to do it	33	20.4
8	Recruiting patients and members of the public with necessary skills	33	20.4
9	Training and support are not available for patients or members of the public	33	20.4
10	Training and support are not available for me as a researcher	27	16.7
11	Concerns about conflicting perspectives	21	13
12	Concerns about the impact of patient and public involvement on research	13	8
13	Don’t know	12	7.4
14	Other people I work with don’t want to involve patients and the public in the design and delivery of our research	9	5.6
15	Concerns about letting go of control	8	4.9
16	I don’t want to involve patients and the public in my research	6	3.7
17	I’m not aware of any barriers	4	2.5
18	There are no barriers	1	0.6
	Other- please specify	27	16.7

Content analysis of the 27 free text responses to Q.15 identified four additional barriers:

1. Institutional and Structural Barriers: *“Lack of practical support at institutional level e.g. governance procedures*,* recruitment templates*,* social media campaign design…” (Senior Lecturer)*.
2. Funding and Resource Constraints: *“No research funding at all – not a lack of funding specific to PPI…” (Research Support Officer)*.
3. Ethics and Governance Challenges: “*For those who receive social welfare*,* for example persons with a disability*,* others have found it challenging to ensure that they were paid as a researcher without their disability payment being affected” (PhD Student).*
4. Researcher Confidence: *“Confidence for me is a big barrier. I have tried to engage as a PPI member myself in other projects to see what it looks like from that perspective….” (Postdoctoral Researcher).*

#### Enablers

Funding to support PPI, good links with patient or community organizations, and PPI workshops and training, were the most widely cited enablers by respondents (See Table 5) (Q.16). These identified enablers were consistent across student and staff populations.

**Table 5:** Enablers for involving patients and members of the public in the design, conduct and dissemination of research

#	Enabler	*N* (*n* = 154)	%
1	Funding to support involving patients or members of the public	112	72.7
2	Good links with patient or community organisations	101	65.6
3	PPI workshops and training	100	65
4	PPI Information and resources	69	44.8
5	One-to-one PPI support	66	42.9
6	Easily accessible patients or members of the public willing to take on this role	62	40.3
7	Previous experience of involving patients and the public in research design and delivery	47	30.5
8	Increased evidence of the impact of PPI on research	46	29.9
9	Other members of my research team are supportive of PPI	37	24
10	There are no enablers	5	3.2
11	I’m not aware of any enablers	3	1.9
	Other- please specify	14	9.1

Content analysis of the 14 free text responses to Q.16 highlighted three additional enablers:

1. Streamlined financial and administrative systems: *“Making it easier to pay my PPI members – this is a huge barrier*,* I have funding*,* but it is very hard to pay them.” (PhD Student)*.
2. Dedicated structures and Networks for capacity building: *“Would love to see something similar [to technicians’ SharePoint group] whereby anyone who has previously included PPI or plans to do some PPI can meet and discuss for an hour.” (Postdoctoral Researcher)*.
3. Attitude and culture change: *“If people stopped using PPI as a buzzword and box ticking exercise and actually did it for themselves.” (PhD Student)*.

#### What should the University do to encourage and develop PPI in research (Q.17)?

The most suggested actions that could be taken by UCC to enable, encourage, and develop PPI in research were to provide practical help to researchers wanting to include PPI in their research, provide opportunities for ‘match making’ between researchers and interested people, and building awareness of PPI. See Table 6.

**Table 6:** What should UCC do to encourage and develop PPI in research (Q.17)?

#	Activity	*N* (*n* = 158)	%
1	Provide practical help to researchers wanting to include PPI in their research	131	83
2	Provide opportunities for ‘match making’ between researchers and interested people	116	73.4
3	Build awareness of PPI	97	61.4
4	Provide training for researchers	95	60.1
5	Provide training for patient/public representatives	94	59.5
6	Develop accredited PPI modules	72	45.6
7	Include patient/public representatives on ethics committees	71	44.9
8	Take PPI into account in job promotions	47	29.7
9	Develop accredited PPI programmes	40	25.3
10	I’m not sure what UCC should do	8	5.1
	Other - please specify	10	6.3

Additional actions emerging from the free text responses (*n* = 10) highlighted the need for: (1) Institutional recognition and integration, (2) Resourcing and funding, (3) Fair treatment and reimbursement, and 3. Cultural change and attitudes.

1. Institutional Recognition and Integration: *“Making PPI more embedded into current UCC systems/procedures would give it the authority it deserves…. for example having a PPI consideration section in the CREC applications and grant/funding writing” (Postdoctoral research)*.
2. Resourcing:*“Provide staff who facilitate PPI management and enable links with researchers and PPIs.” (Research support officer)*.
3. Fair Treatment and Reimbursement: *“Make it easier to reimburse PPI. Also parking – allow PPI contributors to enter staff parking.” (PhD Student)*.
4. Culture and Attitude Change: *“Speaking to senior colleagues about the use of PPI is often brushed off or seen as a ‘fad.’” (Postdoctoral researcher)*.

#### Recommendations

Finally, respondents were asked to provide 3 recommendations of where they would like to see PPI training available for (Q.18). Using content analysis, responses could be broadly categorised into three key areas of PPI training:

1. PPI Recruitment and Facilitation: Respondents most frequently highlighted the early stages of PPI projects as needing further training, particularly how to start, how to recruit PPI contributors, and how to establish a functioning panel. Practical, step-by-step guidance was identified as essential with PPI recruitment difficulties identified as a key challenge. Suggestions included the development of a toolkit to support panel set-up, and creating diverse, institution-level PPI panels that researchers could access. These panels could be organized by research area (e.g. diabetes) supporting contribution across multiple related projects and disciplines.
2. Managing and communicating with PPI Contributors: Maintaining engagement and building effective relationships after recruitment was a key training priority. Respondents emphasised that researchers often lack confidence in communicating contributors from diverse or vulnerable groups and in managing conflicting expectations between researchers and PPI contributors. Training in conflict management, inclusive practice, and respectful negotiation were all identified as important reflecting a broader need for training in communications and relationship management.
3. Sourcing Funding for PPI Activities: Guidance and training in grant writing, and developing strong funding proposals was seen as essential for ensuring that PPI activity is sustainable and appropriately resourced. Respondents also noted that existing resources and guidance on funding can be difficult to find/access. The introduction of simple, information-based awareness campaigns to signpost researchers towards existing materials and services, as well as the provision of more direct, practical training on how to secure financial support for PPI initiatives were suggestions in this area.

## Discussion

Although the importance of PPI in health and social care research is widely acknowledged, it has yet to become routine practice [4]. The current study aimed to answer three core questions: (1) What are UCC staff and students’ understandings of PPI? (2) What are staff and students’ experiences of conducting PPI in UCC? and (3) What are the barriers to PPI, and what should UCC do to develop PPI in research?

### PPI understanding and experiences

Study respondents demonstrated relatively strong understanding of PPI, with almost 70% rating their knowledge as reasonable, good, or very good. This was reinforced by their ability to correctly identify authentic examples of PPI (e.g. involving members of the public in steering committees or research priority setting) while correctly identifying activities which do not constitute PPI. Prior research from the UK and Australia has highlighted that such an understanding is critical for supporting PPI adoption and fundamental to building effective involvement relationships [13–15]. These findings can also be compared to similar survey studies conducted in Ireland which similarly identified two thirds of their respondents having ‘good’ or ‘very good’ understandings of PPI [5].

Despite encouraging levels of PPI knowledge, only half of those familiar with PPI reported having implemented it in their research aligning with similar surveys on researchers’ experiences. For instance, a 2023 study within a European precision oncology project identified a sizeable gap between researchers awareness of PPI and actual engagement in PPI activities [16], while a separate study exploring doctoral nursing students’ knowledge and experiences of PPI identified a gap between students’ theoretical understandings of PPI and practical involvement experience [17].

Although respondents in the current survey reported PPI activity across multiple stages of research, most involvement occurred during study recruitment and design, with fewer examples at later stages such as data analysis and implementation. This trend is consistent with previous evidence. For example, the 2015 RAPPORT study, which evaluated PPI across applied health research projects in the UK, found that PPI involvement was most common at early research stages as opposed to later research stages [18]. This pattern highlights the need for stronger integration of PPI across the full research cycle.

### PPI barriers and areas for future training

In this study, funding, time, and concerns about tokenistic engagement were cited as the main barriers to adopting a PPI approach to research, consistent with a wider body of evidence identifying similar challenges.

Time has been highlighted in previous studies as a pervasive challenge [5, 15]. A previous U.K semi- structured interview study exploring researchers’ experiences and perceptions of PPI describes the significant administrative workload associated with PPI and the absence of dedicated institutional support to manage it [19]. Researchers noted that involvement work often diverted time and effort away from other core activities, raising questions about the sustainability and value placed on PPI.

Funding-related barriers are also evident in several studies, including similar studies carried out in Ireland. A recent Health Research Board paper identified funding and lack thereof as a key barrier to implementing PPI [5]. A realist evaluation conducted to explore how embedded patient PPI is within mainstream health research in England pointed to complexities around payment mechanisms, including difficulties faced by contributors receiving social welfare, while some contributors themselves expressed dissatisfaction with remuneration levels [14]. Similar to our findings, other studies have highlighted concerns about the lack of financial support and infrastructure for PPI, noting that sustainable involvement requires not only money, but also dedicated space, skill development, and institutional coordination [19] and expressed concerns about the sustainability of PPI when funding was limited, particularly where partners could not be adequately compensated for their time and input [17].

Concerns about tokenistic engagement was also identified as a barrier, along with a strong appetite for practical PPI support. This aligns with wider evidence from UK research showing that PPI training raises awareness, shifts attitudes, and builds the skills needed for meaningful involvement [20, 21]. Despite its value, PPI remains largely absent from the traditional curriculum of researchers even though many frameworks and funders highlight it as a priority [22]. In this study, respondents identified three priority areas for future PPI training: PPI recruitment and facilitation, managing and communicating with PPI contributors, and sourcing funding for PPI activities. Respondents also noted a lack of confidence in communicating effectively with contributors from diverse groups, highlighting the importance of practical, skills-based training. This approach is supported by previous UK and European research which has also emphasised the importance of focusing training initiative on “soft” skills such as communication, empathy, and relationship-building to ensure meaningful involvement [16, 19]. At UCC, the findings of this survey have already informed the development of new initiatives, including, the development of a postgraduate PPI module, payment policy, and additional funding for PPI including at undergraduate level.

### Refining PPI terminology and suggestions for future research

In the initial draft of this paper, the term ‘PPI contributor’ was used to describe the involvement of individuals with lived experience in the research process. However, in subsequent group discussions, it was noted that the term ‘PPI contributor’ did not fully capture their involvement in this project. It was agreed that the term ‘PPI partner’ better represented their collaborative and equal role, and this term is used throughout this manuscript.

Our PPI partners recognised the lack of research on their needs, preferences, and experiences as PPI partners engaging in the research process, highlighting a potential gap in the research literature. Given this, the development of a national PPI partner survey was identified as a priority for future research in this area. Additionally, although a strong PPI partnership was present in this study, we recognise that marginalised groups are often unrepresented in such initiatives. Future research should focus on engaging an even broader range and number of PPI partners to ensure perspectives are fully captured.

### Limitations

These findings should be interpreted with caution considering potential sampling bias. As PPI is still an emerging area in Ireland, it is likely that individuals already aware of, or experienced in, PPI activities were more inclined to participate in the survey. The sample was also disproportionately weighted towards the College of Medicine and Health (COMH), which accounted for over half of the responses. This overrepresentation may reflect the more established emphasis on PPI within health and social care research in the COMH, recent institutional efforts within the COMH to embed PPI practices, as well as formal mandates for PPI in funding calls in these contexts (e.g. Health Research Board). Consequently, the perspectives of staff and students from other disciplines may be underrepresented. Thus, data highlights a need for increased training and PPI awareness in other colleges across UCC. As the overall sample constitutes only a small fraction of the university community, the generalisability of these findings across UCC must be considered limited.

Further limitations of this study are the high level of attrition, item non-response, and the fact that the established goal of 350 responses was not reached. Although 289 individuals accessed the survey, completion rates varied substantially across items (e.g., 272 responses to item 1 versus 174 to item 13), with 160 respondents completing the survey in full. While the survey design allowed respondents to answer items independently and did not require full completion, thereby reducing the impact of missing data on individual analyses, the fluctuating response rates nonetheless raise concerns about data completeness and potential response bias. This pattern suggests that later items may have been disproportionately influenced by a smaller, potentially more motivated subset of participants.

Beyond establishing content validity, the survey did not undergo additional formal validation procedure to test construct validity, content validity or reliability, therefore this survey should therefore be interpreted as an exploratory instrument designed to provide initial baseline data rather than a fully validated measurement tool. Finally, incorporating findings from qualitative surveys of barriers and enablers more explicitly in future research could strengthen survey development and improve the comprehensiveness of the instrument. Despite these limitations, the dataset remains valuable and provides meaningful insights into staff and students’ understandings and experiences of PPI.

## Conclusion

This study demonstrates that while UCC staff and students report a generally strong understanding of PPI, practical implementation remains limited and constrained by barriers such as time, funding, and concerns about tokenism. Addressing these challenges requires institutional commitment, resources, and cultural change. A future study of this kind may help to provide insights into the progression of PPI implementation and practice in UCC. Prioritising targeted training alongside structural supports offers clear opportunities to strengthen capacity and embed meaningful PPI within the university research environment.

## Electronic Supplementary Material

Below is the link to the electronic supplementary material.

Supplementary Material 1

Supplementary Material 2

## Data Availability

The data used in this study cannot be shared publicly due to the conditions of the original ethical approval, which limited access to the research team only. Because data sharing was not included in the ethics application, retrospective approval cannot be obtained. All analyses are based on the full dataset, and all relevant results are reported in the manuscript.

## Abbreviations

PPI: Public and Patient Involvement
UCC: University College Cork
SREC: Social Research Ethics Committee

## References

[1] National Institute for Health Research (NIHR). National Institute for Health Research (NIHR) [Internet]. 2024 [cited 2025 Jul 23]. Briefing notes for researchers - public involvement in NHS, health and social care research. Available from: https://www.nihr.ac.uk/briefing-notes-researchers-public-involvement-nhs-health-and-social-care-research.

[2] Brett J, Staniszewska S, Mockford C, Herron-Marx S, Hughes J, Tysall C, et al. Mapping the impact of patient and public involvement on health and social care research: a systematic review. Health Expect. 2014;17(5):637–50. 22809132 10.1111/j.1369-7625.2012.00795.x PMC5060910

[3] Greenhalgh T, Hinton L, Finlay T, Macfarlane A, Fahy N, Clyde B, et al. Frameworks for supporting patient and public involvement in research: Systematic review and co-design pilot. Health Expect. 2019;22(4):785–801. 10.1111/hex.12888 . PubMed PMID: 31012259. 31012259 10.1111/hex.12888 PMC6737756

[4] Lang I, King A, Jenkins G, Boddy K, Khan Z, Liabo K. How common is Patient and Public Involvement (PPI)? Cross-sectional analysis of frequency of PPI reporting in health research papers and associations with methods, funding sources and other factors. BMJ Open. 2022;12(5). 10.1136/bmjopen-2022-063356. PubMed PMID: 35613748. 10.1136/bmjopen-2022-063356 PMC9131100 35613748

[5] Mizzoni C, Lord S, Cahill A, Cody A. Researcher and Funder perspectives on research funders’ support for Public and Patient Involvement in Research (PPI). HRB Open Res. 2025;8:97. 10.12688/hrbopenres.14178.1.

[6] Ocloo J, Matthews R. From tokenism to empowerment: progressing patient and public involvement in healthcare improvement. BMJ Qual Saf. 2016;bmjqs-2015-004839. 10.1136/bmjqs-2015-004839 PMC4975844 26993640

[7] Zapf M, Refaeil N, de Leon BA. Comprehensive Capacity Development: Moving Beyond Training as the Default. J Peacebuilding Dev. 2019;14(3):340–4. 10.1177/1542316619871231.

[8] Markaki A, Malhotra S, Billings R, Theus L. Training needs assessment: tool utilization and global impact. BMC Med Educ. 2021;21(1). 10.1186/s12909-021-02748-y. PubMed PMID: 34059018. 10.1186/s12909-021-02748-y PMC8167940 34059018

[9] PPI Ignite Network. PPI Ignite Network [Internet]. 2025 [cited 2025 Jul 24]. Available from: https://ppinetwork.ie/about-us/.

[10] Kmetty Z, Stefkovics Á. Assessing the effect of questionnaire design on unit and item-nonresponse: evidence from an online experiment. Int J Soc Res Methodol. 2022;25(5):659–72. 10.1080/13645579.2021.1929714.

[11] Krippendorff Klaus. Content analysis: an introduction to its methodology. SAGE; 2019. p. 453.

[12] Gitonga I, Keegan D, Kay Livingston M, Partner Paula Leocadio P, John S, Morejon Hernandez Y. A friendly guide to Equality, Diversity & Inclusion (EDI) in Public and Patient Involvement (PPI) a friendly guide to equality, diversity and inclusion.

[13] Miller CL, Mott K, Cousins M, Miller S, Johnson A, Lawson T, et al. Integrating consumer engagement in health and medical research - an Australian framework. Health Res Policy Syst BioMed Cent Ltd. 2017. 10.1186/s12961-017-0171-2 . PubMed PMID: 28187772. 10.1186/s12961-017-0171-2 PMC5303288 28187772

[14] Wilson P, Mathie E, Poland F, Keenan J, Howe A, Munday D, et al. How embedded is public involvement in mainstream health research in England a decade after policy implementation? A realist evaluation. J Health Serv Res Policy. 2018;23(2):98–106. doi:10.1177/1355819617750688 PubMed PMID: 29653504. 29653504 10.1177/1355819617750688 PMC5901416

[15] McKenzie A, Alpers K, Heyworth J, Phuong C, Hanley B. Consumer and community involvement in health and medical research: evaluation by online survey of Australian training workshops for researchers. Res Involv Engagem. 2016;2(1). 10.1186/s40900-016-0030-2. 10.1186/s40900-016-0030-2 PMC5611564 29062517

[16] Mosconi P, Colombo C, Paletta P, Gangeri L, Pellegrini C, Garralda E, et al. Public and patient involvement: a survey on knowledge, experience and opinions among researchers within a precision oncology European project. BMC Cancer. 2023;23(1). 10.1186/s12885-023-11262-x . PubMed PMID: 37648965. 10.1186/s12885-023-11262-x PMC10470190 37648965

[17] Tanay MA, Diez de los Rios, de la Serna C, Boland V, Lopes AMDS, Wingfield K, Chircop D et al. Patient and public involvement in research: reflections and experiences of doctoral cancer nurse researchers in Europe. Euro J Oncol Nurs. 2023;64. 10.1016/j.ejon.2023.102351 PubMed PMID: 37290166. 10.1016/j.ejon.2023.102351 37290166

[18] Wilson P, Mathie E, Keenan J, McNeilly E, Goodman C, Howe A et al. ReseArch with patient and public involvement: a realist evaluation: the RAPPORT study. 2015. 26378332

[19] Boylan AM, Locock L, Thomson R, Staniszewska S. About sixty per cent I want to do it: Health researchers’ attitudes to, and experiences of, patient and public involvement (PPI)—A qualitative interview study. Health Expect. 2019;22(4):721–30. 10.1111/hex.12883 . PubMed PMID: 30927334. 30927334 10.1111/hex.12883 PMC6737750

[20] Dudley L, Gamble C, Preston J, Buck D, Hanley B, Williamson P et al. What difference does patient and public involvement make and what are its pathways to impact? Qualitative study of patients and researchers from a cohort of randomised clinical trials. PLoS One [Internet]. 2015;10(6):e0128817. Available from: https://www.internal-pdf://93.103.134.114/Dudley-2015-Whatdifferencedoespatientandp.pdf. 10.1371/journal.pone.0128817 PMC4459695 26053063

[21] Yu R, Hanley B, Denegri S, Ahmed J, McNally NJ. Evaluation of a patient and public involvement training programme for researchers at a large biomedical research centre in the UK. BMJ Open. 2021;11(8). 10.1136/bmjopen-2020-047995 . PubMed PMID: 34385250. 10.1136/bmjopen-2020-047995 PMC8362711 34385250

[22] de Wit M, Beurskens A, Piškur B, Stoffers E, Moser A. Preparing researchers for patient and public involvement in scientific research: Development of a hands-on learning approach through action research. Health Expect. 2018;21(4):752–63. 10.1111/hex.12671 . PubMed PMID: 29418053. 29418053 10.1111/hex.12671 PMC6117481

