# Supplementary material for: Exploring staff and students’ understanding and experience of Public and Patient Involvement (PPI) in an Irish University
Source: Res Involv Engagem. 2026 Jun 23;12:101. doi: 10.1186/s40900-026-00896-3 (PMC13292565; doi:10.1186/s40900-026-00896-3)
Supplement: Supplementary file 1 — Supplementary Material 1 [file 40900_2026_896_MOESM1_ESM.docx]

**Supplementary Table 1:** Summary of Survey Instrument Questions.

| Question  Number | Question |
| --- | --- |
|  | Section 1: Demographics |
| 1 | What is your current role? |
| 2 | What do you identify as? |
| 3 | Please indicate your primary affiliation. |
|  | Section 2: Understanding of PPI in Research |
| 4 | How would you rate your understanding of PPI? |
| 5 | Can you describe what PPI is, in your own terms? |
| 6 | What actions in the list below do you consider to be PPI? (Select all that apply) |
|  | Section 3: Experience |
| 7 | Do you have any experience with involving members of the public, patients or carers in the design or delivery of your research? |
| 8 | How would you rate your experience of involving members of the public, patients or carers in the design or delivery of your research? |
| 9 | Please select the stages where you have involved members of the public, patients or carers in your research (please tick all that apply) |
| 10 | What was your motivation for involving patients or members of the public in the design and delivery of your research? (*Please tick all that apply)* |
| 11 | Have you received any training for involving patients or members of the public in your research? |
| 12 | Have you received any advice or support for involving patients or members of the public in your research? |
| 13 | Are you interested in involving patients or members of the public in the design and delivery of your research in the future? |
| 14 | If you have not yet considered involving patients or members of the public in the design and delivery of your research, please outline the reason why |
|  | Section 4: Barriers and Enablers |
| 15 | What are the barriers to involving patients and members of the public in the design, conduct and dissemination of your research? (Please tick all that apply) |
| 16 | What would enable you to involve patients and members of the public in the design, conduct and dissemination of your research? (Please tick all that apply) |
| 17 | What should UCC do to encourage and develop PPI in research? (Please tick all that apply) |
| 18 | Suggest three areas or topics that you would like to see PPI training available for in the future? |
