# Supplementary material for: Exploring staff and students’ understanding and experience of Public and Patient Involvement (PPI) in an Irish University
Source: Res Involv Engagem. 2026 Jun 23;12:101. doi: 10.1186/s40900-026-00896-3 (PMC13292565; doi:10.1186/s40900-026-00896-3)
Supplement: Supplementary file 2 — Supplementary Material 2 [file 40900_2026_896_MOESM2_ESM.docx]

# **Appendix A: Copy of Survey Instrument**

**PPI Ignite Network @ UCC** **Survey 2023:**

**Public and Patient Involvement (PPI) in Research**

Instructions

Public and Patient Involvement (PPI) involves patients, members of the public, researchers and organisations working in partnership to decide what to research and how best to carry out that research together. The following questions will ask you about your understanding and experience of public and patient involvement (PPI) in research at University College Cork.

Please select one answer only for each question unless specified otherwise.

I am aged 18 years or over ☐

Section 1: DemographicsQ1. What is your current role? 
 
*Please select the most senior role that applies:*

Undergraduate Student

Masters Student

PhD student

Research Administrator

General Administrator

School/ Department Manager

Research Assistant

Research Associate

Research Support Officer

Postdoctoral Researcher

Research Fellow

Lecturer

Senior Lecturer

Professor

Other (please specify) _________________________________________________

**Q2. What do you identify as?**

Male

Female

Prefer not to say

Other

**Q3. Please indicate your primary affiliation.**

College of Arts, Celtic Studies, and Social Science

College of Medicine and Health

College of Business and Law

College of Science, Engineering and Food Science

Other (please specify)_________________________________________________________

Section 2: Understanding of PPI in research.

Q4. How would you rate your understanding of PPI?

Very good

Good

Reasonable

Limited

No understanding

**Q5. Can you describe what PPI is, in your own terms?**

__________________________________________________________________________________________________________________________________________________________________________________________________________________________________________________________________________________________________________________________________________________________________________________________________________________________________________________________________

Q6. What actions in the list below do you consider to be PPI? (Select all that apply)

Recruiting study participants to take part in a study.

Inviting members of the public, patients or carers to sit on a study/ research group steering committee.

Conducting qualitative interviews, where researchers interview study participants.

Naming a patient or a member of the public/community group/ patient organisation as a co-application on a grant application.

Working with members of the public and other stakeholders to set research priorities.

Inviting the public to attend information evening to raise awareness about research.

Visiting schools to make research and science interesting with school students.

Researchers communicating research to the public via TV, radio, print and social media.

Don’t know

Section 3: Experience

What is PPI?

Public, Patient and Carer Involvement (PPI) is research carried out ‘with’ or ‘by’ members of the public rather than ‘to’, ‘about’ or ‘for’ them. PPI is a mechanism for people to contribute to the research process, for example by identifying what research is important, checking that the language of materials for participants in a study is appropriate and understandable or participation in data analysis or in an oversight group for a study.


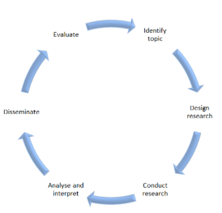


It can also involve things like designing and refining the research plan, helping with the recruitment of participants and other aspects of the delivery of the study, and publicising the outputs. Input can be at any stage of the research cycle but will have the greatest impact if it comes early.

Q7. Do you have any experience with involving members of the public, patients or carers in the design or delivery of your research?

Yes, I always involve members of the public, patients or carers in as many aspects of my research as possible.

Yes, I have sometimes involved members of the public, patients or carers in one or two aspects of my research.

No, I have not yet involved members of the public, patients or carers in my research. (Skip to question 13)

Q8. How would you rate your experience of involving members of the public, patients or carers in the design or delivery of your research?

Very satisfied

Somewhat satisfied

Neither satisfied or dissatisfied

Somewhat dissatisfied

Very dissatisfied

Q9. Please select the stages where you have involved members of the public, patients or carers in your research (please tick all that apply)

Agenda setting and funding e.g. generating ideas, identifying research topics, prioritising research topics, funding applications, protocol preparation

Study design and procedures e.g. developing research instruments, choosing primary research outcomes, finalising research protocols

Study recruitment e.g. developing research subject recruitment procedures and materials

Data collection e.g public or patient administered data collection such as interviews or surveys

Data analysis e.g. interpretation of findings, external review

Dissemination e.g. presentations, manuscripts, social media, plans for future studies

Implementation e.g. developing decision aid tools, developing clinical practice guidelines

Evaluation e.g. evaluation of process measures, adherence and uptake of interventions, plans for future research

Other – please specify________________________________________________________

Q10 What was your motivation for involving patients or members of the public in the design and delivery of your research? (*Please tick all that apply)*

Requirement for research funders

Requirement of the principal investigator or project lead

Specific issue to be addressed - please provide details_______________________________

I have involved patients and/or members of the general public previously

A patient or member of the public offered their services

I thought it was a good idea/right thing to do

To improve the quality of the research

I don't know what the motivation was for involving patients or members of the public

Other- please specify __________________________________________________________

**Q11. Have you received any training for involving patients or members of the public in your research?**

Yes- please provide details ____________________________________________________

No

**Q12. Have you received any advice or support for involving patients or members of the public in your research?**

Yes- please provide details ____________________________________________________

No

**Q13. Are you interested in involving patients or members of the public in the design and delivery of your research in the future?**

Yes, involving patient or public could benefit my research and I plan to develop it

Maybe, if I can get support

No, I can't see the benefit

**Q14. If you have not yet considered involving patients or members of the public in the design and delivery of your research, please outline the reason why.**

__________________________________________________________________________________________________________________________________________________________________________________________________________________________________________________________________________________________________________________________________________________________________________________________________________________________

**Section 4: Barriers and enablers**

**Q15. What are the barriers to involving patients and members of the public in the design, conduct and dissemination of your research? (Please tick all that apply)**

Time

Funding

I don't know how to do it

I don't want to involve patients and the public in my research

Other people I work with don't want to involve patients and the public in the design and delivery of our research

I have found it difficult to pay PPI contributors

Recruiting patients and members of the public who are interested

Recruiting patients and members of the public with necessary skills

Training and support is not available for me as a researcher

Training and support is not available for patients or members of the public

Concerns about the impact of patient and public involvement on research

Concerns about tokenistic engagement

Concerns about not being able to meet patient or public expectations

Concerns about letting go of control

Concerns about conflicting perspectives

I’m not aware of any barriers

There are no barriers

Don’t know

Other- please specify_____________________________________________________

**Q16. What would enable you to involve patients and members of the public in the design, conduct and dissemination of your research? (Please tick all that apply)**

Funding to support involving patients or members of the public

PPI workshops and training

One-to-one PPI support

Good links with patient or community organisations

Easily accessible patients or members of the public willing to take on this role

PPI Information and resources

Increased evidence of the impact of PPI on research

Previous experience of involving patients and the public in research design and delivery

Other members of my research team are supportive of PPI

I’m not aware of any enablers

There are no enablers

Other- please specify__________________________________________________________

**Q17. What should UCC do to encourage and develop PPI in research? (Please tick all that apply)**

Build awareness of PPI

Provide practical help to researchers wanting to include PPI in their research

Provide opportunities for 'match making' between researchers and interested people

Provide training for patient/public representatives

Provide training for researchers

Develop accredited PPI modules

Develop accredited PPI programmes

Take PPI into account in job promotions

Include patient/public representatives on ethics committees

I’m not sure what UCC should do

Other - please specify__________________________________________________________

**Q18. Suggest three areas or topics that you would like to see PPI training available for in the future?**

1. ___________________________________________________________________________
2. ___________________________________________________________________________
3. ___________________________________________________________________________

**Thank you for taking the time to complete this survey. Your feedback will be invaluable to PPI Ignite @ UCC in terms of developing its programme for the coming year.**

# **Appendix B: Content Analysis of Responses**

**1. Research “With” Not “On” Patients/Public**

Many respondents emphasized that PPI is about conducting research *with* or *by* patients and the public, rather than doing research *about* or *on* them.

*“Research carried out with public or patient involvement.”*

*“Ensuring that research is being done 'with' the public/patients as opposed to 'on' them.”*

*“Doing research WITH those who are impacted by it.”*

**2. Involvement Across All Research Stages**

A common theme was the inclusion of patients/public at every stage of the research process, from idea generation and study design to analysis and dissemination.

*“Public and Patient Involvement at every stage of research.”*

*“Planning, designing and conducting research with input from service users/informal carers/family members affected by conditions.”*

*“Involving the public and end users in research from the beginning and communicating and discussing the research throughout.”*

**3. Collaboration and Partnership**

Respondents highlighted active collaboration between researchers, patients, and the public, often framing it as co-production or partnership.

*“Partnership with members of public, patients and their supporters in designing and carrying out research.”*

*“Researchers and members of the public work in partnership to plan and conduct research.”*

*“Working together in a team with advocates/patients with lived experience on research projects from beginning to end as equal team members.”*

**4.Inclusion of Lived Experience / Patient Perspective**

Many responses stressed the value of experiential knowledge and incorporating patients lived experience to inform research.

*“Using the lived experience of members of the public and/or patients to inform research design, conduct, writing, etc.”*

*“The involvement of individuals with lived experience in the area of a research topic, whereby they are a core contributor to the conceptualization/design of the study.”*

**5. Enhancing Relevance and Impact of Research**

Respondents saw PPI as a mechanism to ensure research addresses real-world needs and is meaningful for participants.

*“Involving the public in better identifying what to research, where the need lies and involving at every stage of research.”*

*“Researchers working in partnership or drawing on the voice/opinions/experiences of the public to produce research and/or interventions which is relevant and applicable to communities.”*

# **Appendix C: Content Analysis of Responses**

If you have not yet considered involving patients or the public in the design and delivery of your research, please outline the reason why.

**1. Perceived Irrelevance to their Role and/or Research Area**

Many respondents explained that PPI does not apply to them because their role does not involve designing or delivering research. Some respondents felt PPI was not relevant to their research, particularly in basic science, engineering, biomedical, or historical research:

*“My research is in basic science, and the potential societal applications may only become apparent many decades in the future.”*

*“I’m a historian – I only care about people who’ve been dead for 500 years.”*

*“My research is fundamental biomedical research and thus it is difficult to foresee how a patient might influence the design.”*

**2. Lack of Awareness or Understanding**

Several participants had not considered PPI because they were unaware of it, had only recently learned about it, or did not fully understand the concept.

*“Only recent understanding of SREC process.”*

*“Don’t understand it.”*

*“I have only become aware of PPI in the past 18 months.”*

*“It just never occurred to me!”*

**3. Structural, Practical and Institutional Barriers**

Respondents cited time, funding, and feasibility as major barriers to including PPI. Small projects, lack of staff support, and difficulty fitting PPI into grant timelines were frequently mentioned.

*“No money.”*

*“Has not been feasible as part of a small project, would not get it done in a timely manner.”*

*“Not always feasible to get PPI involvement when grant writing.”*

*“I need time to buyout/allocate for this, unfortunately my role is quite busy with teaching, admin and research with little support.”*

*“There seems to be slow uptake from the senior members… People just want to have the PPI box ticked.”*

*“Principal investigators did not go down that path.”*

1. **Lack of Training, Support, and Tools**

A number of respondents highlighted that they lacked the training, guidance, or institutional support needed to effectively implement PPI.

*“I lacked the training to do it.”*

*“There is a lack of support in relation to the provision of the necessary toolkits for implementation.”*

*“Never really an option discussed in lectures.”*

5. Future Intentions to embed PPI

Several participants stated that while they had not yet considered PPI, they were open to doing so in future projects.

*“I do plan to do it.”*

*“I have not yet had a good opportunity to do so, but will certainly consider this in the future.”*

*“I am currently considering it.”*
